# Uvéite antérieure révélatrice d'une sarcoïdose

**DOI:** 10.11604/pamj.2015.20.354.4122

**Published:** 2015-04-14

**Authors:** Amal Alouan, Rajae Daoudi

**Affiliations:** 1 Université Mohammed V Souissi, Service d'Ophtalmologie A de l'Hôpital des Spécialités, Centre Hospitalier Universitaire, Rabat, Maroc

**Keywords:** Uvéite antérieure, sarcoïdose, rougeur oculaire, Anterior uveitis revealing sarcoidosis, Anterior uveitis revealing sarcoidosis, eye redness

## Image en medicine

Il s'agit d'une patiente âgée de 40 ans, antécédents de rougeur oculaire à répétition en niveau des deux yeux et qui présente depuis une semaine un ‘il gauche rouge douloureux avec baisse d'acuité visuelle. L'examen ophtalmologique retrouve une acuité visuelle à 3/10 au niveau de l’œil gauche et 8/10 au niveau de l’œil droit. A la lampe à fente, on trouve au niveau de l’œil gauche des précipités rétro-cornéens inférieurs, blanchâtres dites en graisse de mouton, un Tyndall inflammatoire de chambre antérieur 1+ et des synéchies irido-cristalliniennes diffuses. Au niveau de l’œil droit, pas d'inflammation active mais présence de synéchies irido-cristalliniennes qui ne relâchent pas après dilatation (stigmate d'uvéite antérieure). La sarcoïdose est une granulomatose systémique d’étiologie inconnue caractérisée par la formation de granulomes immunitaires au niveau des organes atteints. L'atteinte oculaire est retrouvée chez 25% à 50% des cas et peut être inaugurale dans 20% des cas. Presque tous les éléments du globe, des annexes et de l'orbite peuvent être touchés. Typiquement, l'uvéite antérieure est chronique, granulomateuse et synéchiante. le traitement de référence est la corticothérapie qu'il s'agisse de forme oculaire ou systémique. 3 diagnostics différentiels de l'uvéite antérieure granulomateuse: la tuberculose, la syphilis et maladie de Lyme.

**Figure 1:**
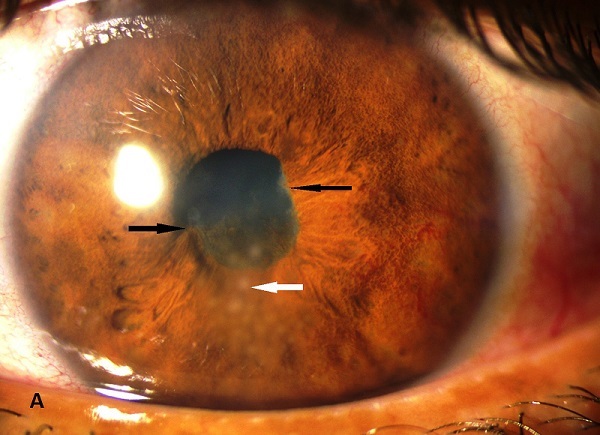
Précipités rétro cornéens granulomateux en graisse de mouton (flèche blanche) Synéchies irido-cristalliniens (flèches noires)

